# Subcellular Interactions during Vascular Morphogenesis in 3D Cocultures between Endothelial Cells and Fibroblasts

**DOI:** 10.3390/ijms18122590

**Published:** 2017-12-01

**Authors:** Sabine Kaessmeyer, Julia Sehl, Maneenooch Khiao In, Roswitha Merle, Ken Richardson, Johanna Plendl

**Affiliations:** 1 Department of Veterinary Medicine, Institute of Veterinary Anatomy, Freie Universitaet Berlin, Koserstraße 20, 14195 Berlin, Germany; Julia.Sehl@fli.de (J.S.); Maneenooch.Khiao-In@fu-berlin.de (M.K.I.); johanna.plendl@fu-berlin.de (J.P.); 2 Department of Veterinary Medicine, Institute of Veterinary Epidemiology and Biostatistics, Freie Universitaet Berlin, Koenigsweg 67, 14163 Berlin, Germany; roswitha.merle@fu-berlin.de; 3 College of Veterinary Medicine, School of Veterinary and Life Sciences, Murdoch University, Murdoch, WA 6150, Australia; K.Richardson@murdoch.edu.au

**Keywords:** endothelial cells, fibroblasts, 3D cocultures, angiogenesis, extracellular matrix, collagen, fibronectin, laminin, morphology, ultrastructure

## Abstract

Background: Increasing the complexity of in vitro systems to mimic three-dimensional tissues and the cellular interactions within them will increase the reliability of data that were previously collected with in vitro systems. In vivo vascularization is based on complex and clearly defined cell–matrix and cell–cell interactions, where the extracellular matrix (ECM) seems to play a very important role. The aim of this study was to monitor and visualize the subcellular and molecular interactions between endothelial cells (ECs), fibroblasts, and their surrounding microenvironment during vascular morphogenesis in a three-dimensional coculture model. Methods: Quantitative and qualitative analyses during the generation of a coculture tissue construct consisting of endothelial cells and fibroblasts were done using transmission electron microscopy and immunohistochemistry. Results: Dynamic interactions were found in cocultures between ECs, between fibroblasts (FBs), between ECs and FBs, and between the cells and the ECM. Microvesicles were involved in intercellular information transfer. FBs took an active and physical part in the angiogenesis process. The ECM deposited by the cells triggered endothelial angiogenic activity. Capillary-like tubular structures developed and matured. Moreover, some ECM assembled into a basement membrane (BM) having three different layers equivalent to those seen in vivo. Finally, the three-dimensional in vitro construct mirrored the topography of histological tissue sections. Conclusion: Our results visualize the importance of the physical contact between all cellular and acellular components of the cocultures.

## 1. Introduction

Increasing the complexity of in vitro systems to mimic three-dimensional tissues and the cellular interactions within them will increase the reliability of data that were previously collected with in vitro systems. The development of these in vitro systems requires models that display maximum resemblance to the situations found in tissues in vivo. Adequate morphogenesis and cell functions depend on close interactions between cells as well as between cells and their matrix, because both types of interaction are critical for the organization of a microenvironment in vitro that is as close to the in vivo situation as possible. Without the latter, cells lose their organotypical characteristics and transdifferentiate [1,2].

In vivo, capillaries are embedded in a microenvironment that consists of the extracellular matrix (ECM) and of cellular components including fibroblasts (FBs) as well as immune cells. The ECM is a complex, noncellular network composed of distinct components that is found in two different locations, i.e., in the interstitium, as interstitial ECM and in association with all epithelia and endothelia, as the basement membrane (BM) [3]. In vivo, the ECM components are mainly synthesized by FBs, which, among others, secrete various collagens, fibronectin, and heparin sulfate proteoglycans [4,5]. The development of blood vessels, which is a requirement for growth and regeneration, depends on a highly structured communication of endothelial cells (ECs) with their surrounding tissue. In vivo vascularization is based on complex cell–matrix and cell–cell interactions, in which the ECM and the FBs seem to play a very important role [6,7]. Blood vessels either develop de novo by the process of vasculogenesis, or they arise by angiogenesis, where new capillaries grow from already existing ones. Vasculogenesis is the determination and differentiation of endothelial precursor cells that arrange themselves into aggregates of cells and then create a simple network of tubes having a lumen [8]. Angiogenesis appears to occur by two mechanisms, namely non-sprouting (intussusceptive) and sprouting angiogenesis [9]. During intussusception, endothelial protrusions of opposing capillary walls extend towards each other and fuse creating an interendothelial contact [8].

Sprouting angiogenesis has been a recent focus of intense research. It is a process having many sequential hierarchical steps that require the close interaction of ECs with both acellular and cellular components of the surrounding tissue, including FBs [4]. In fact, FBs exert a significant role in angiogenesis [5]. These cells synthesize angiogenic growth factors that trigger sprouting angiogenesis. These factors include vascular endothelial growth factor (VEGF-A), transforming growth factor-β (TGF-β), and platelet-derived growth factor (PDGF) [4,5,10]. Angiogenic stimuli activate the ECs to migrate into the avascular tissue [11]. ECs express VEGF-receptor 2 that responds to the VEGF-A gradient and binds to the growth factor. Once an angiogenic stimulus occurs, metalloproteinases (MMPs) break down the basement membrane (BM) of the blood vessel, mainly near the trigger sites [12]. During sprouting, ECs are triggered by the VEGF-R2–VEGF-A reaction to temporarily transform into migrating tip cells. These cells are polarized and have well-developed filopodia that enable them to interact with the ECM via integrins. Integrins (primarily alphaVß3) on the ECs’ filopodia surface have an adhesive function during endothelial migration [11]. ECM proteins, mainly synthesized by FBs, are important for adhesion and migratory processes of the endothelial tip cells and therefore promote angiogenesis [3,6,13,14]. The endothelial tip cells traverse into the surrounding avascular extracellular matrix towards the angiogenic stimulus [15]. To enable this process, the MMPs form tunnels in the ECM to facilitate endothelial migration [16]. Membrane-type 1 matrix metalloproteinases (MT1-MMP), selectively expressed by the endothelial tip cells, are responsible for most of the proteolysis of the ECM [15,17]. Endothelial stalk cells follow the tip cells into the ECM where they proliferate and elongate the developing capillary sprout, as well as establish its internal lumen. The tubular lumen is formed by intraendothelial vacuoles that fuse. For the development of the vacuoles, MT1-MMP and the integrins alphaVß3 and alpha5ß1 play important roles [18]. In addition, FB-derived matrix proteins, i.e., collagen I and fibronectin, are necessary for tube formation [5,6]. Tight and adherens cell junctions are established between the stalk cells of the newly built tube, and, consequently, a new vessel arises.

Due to their roles in cell–matrix interactions and especially in matrix remodeling, FBs are crucial in vascular development through transmitting biochemical signals and mechanical forces that affect endothelial cell survival, cell shape, and cell orientation [19]. In cooperation with endothelial stalk cells, surrounding FBs synthesize basement membrane proteins, namely laminin, collagen IV, perlecan, nidogen, collagen XVIII, and fibronectin. The BM envelops and stabilizes the newly developing capillary sprout, serving as an acellular barrier against the capillaries microenvironment and ensuring the correct polarity of ECs [12]. It typically consists of an electron-dense layer (lamina densa) separated from the cellular plasma membrane by an electron-lucent layer (lamina lucida). The outermost third sheet of the BM is a less dense but thicker layer of diffuse ECM that constitutes the lamina fibroreticularis [20]. Maturation, stabilization, and remodeling of the dynamic capillary structures follow initial angiogenesis [21]. As tubules mature, their ECs transform into quiescent phalanx cells [22].

While the molecular aspects of angiogenesis, including signal molecules and their specific receptors, have been studied in great detail, hardly any attention has been given to the physical interaction, communication, and exchange of information between cells or between cells and their immediate surroundings.

The aim of this study was to monitor and visualize the cellular events and interactions between ECs, FBs, and their surrounding microenvironment during vascular morphogenesis in a three-dimensional coculture model. Moreover, the ECM proteins laminin, fibronectin, and collagen III, which are known to be significant at different steps of angiogenesis, were examined morphometrically.

## 2. Results

### 2.1. Ultrastructural Observations of Vascular Development and Extracellular Matrix Organization over the Timeline from 5 to 20 Days in Cocultures of Endothelial Cells with Fibroblasts and in Monocultures

#### 2.1.1. The Cells: Filopodia, Vesicles, and Caveolae for Intercellular Communication and Lumen Formation

Cocultured FBs and ECs could be distinguished in vitro by typical morphological and ultrastructural features. Over the timeline of 20 days, the oval to spindle-shaped FBs contained a characteristically large amount of rough endoplasmic reticulum (rER) and formed extensive filopodial protrusions. In addition, fine communicating protrusions bridged short distances between neighboring FBs. In the late stages of monitoring (20 days), individual fibroblastic cells with smaller amounts of rER were detected, indicating their differentiation into fibrocytes.

**Figure 1:**
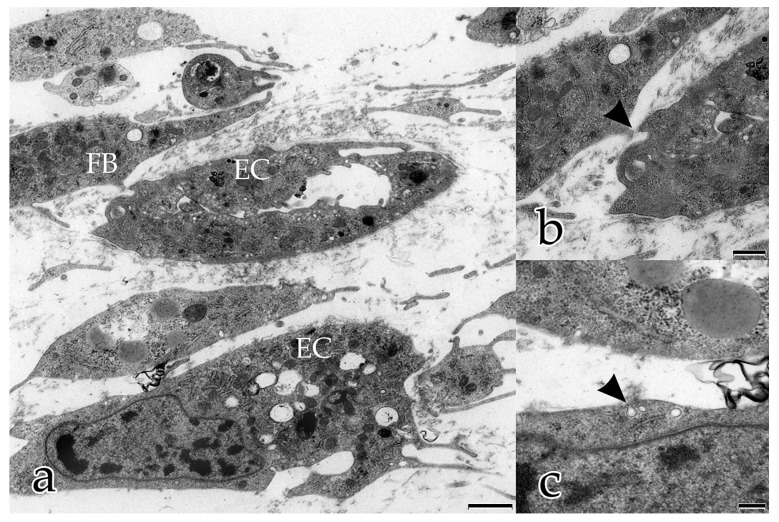
Coculture of ECs with FBs after 5 days: (**a**–**c**) intercellular communication between FBs, between ECs, and between FBs and ECs; (**b**), magnification of (**a**) EC and FB interaction by small protrusions (arrowhead); (**c**) magnification of (**a**) EC with small vesicles and caveolae extruding and/or ingesting extracellular material (arrowhead). (**a**) Bar: 1.000 nm, (**b**) Bar: 500 nm, (**c**) Bar: 250 nm.

Over the same timeline, ECs underwent massive morphological changes during in vitro angiogenesis. After 5 days, the shape of ECs varied greatly, and some individual cells had a typical curved cellular body shape, suggestive of later assembling into tubular structures (Figure 1a). Most ECs had developed multipolar tentacle-like protrusions, having morphological characteristics of endothelial tip cells. These cells were in contact either with neighboring cells or with the ECM. Their nuclei were characterized by a large amount of heterochromatin and their cytoplasm was rich in organelles. Distinctive EC features, such as abundant small vesicles in the cytoplasm near the cell membrane and the presence of caveolae at or within the cell membrane, suggested that they extruded intracellular material (Figure 1a–c), ingested extracellular material by pinocytosis, or both. This (potential) form of intercellular communication was seen frequently between FBs as well as between ECs, but also between FBs and ECs (Figure 1a–c).

**Figure 2:**
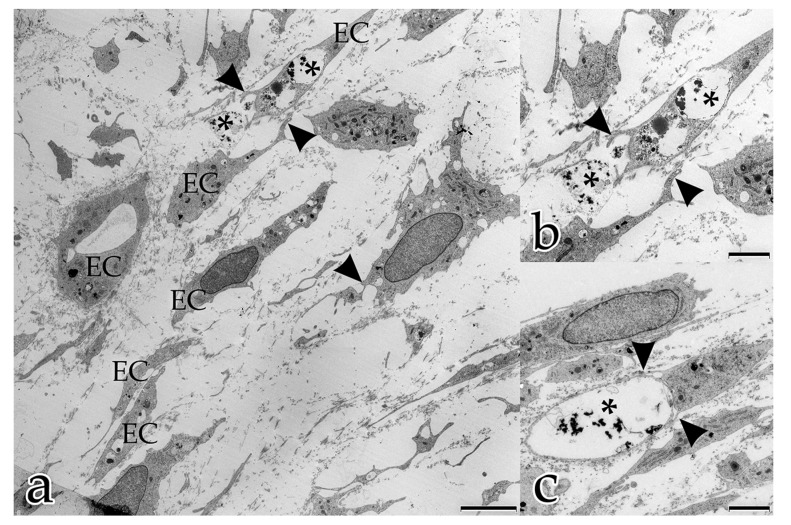
Coculture of ECs with FBs after 10 days: (**a**–**c**) ECs organize into chains that align with the main orientation of the ECM. Cells are connected to each other by their polar cellular protrusions (arrowheads); (**a**) angiogenic ECs use the ECM as guiding structures to line up into chains; (**b**) magnification of (**a**); (**c**) endothelial stalk cells fuse into a tube, with lumenization by fusion of intracellular and extracellular vacuoles, respectively (asterisks). (**a**) Bar: 5.000 nm, (**b**,**c**) Bar: 2.500 nm.

Day 10: vacuoles within the cytoplasm of ECs had often fused into several larger units where, in extreme cases, the entire cytoplasm was occupied by a single massive vacuole. Endothelial cells were also observed to extrude these vacuolar structures into the intercellular space between neighboring ECs (Figure 2a). The ECs that had not already established a tube organized themselves into chains following the principal orientation of the ECM, and were connected to each other by their polar protrusions (Figure 2b). The filopodia of those ECs encircled extracellular vacuoles (Figure 2c). Subsequently, the cells of the chains appeared to fuse into a tube, indicating the presence of endothelial stalk cells. While the number of small intracellular vesicles near the cell membrane had decreased (compared to the observations at day 5), larger vacuolar structures and lysosomes now filled the cells. ECs that were already part of a tubular structure, had a finely granulated cytoplasm with small vesicles, few vacuoles, and numerous membrane-bound caveolae. Vacuoles and parts of the cellular membranes were present in the tubules’ lumina.

Day 14: sprouting and tubular establishment from both intra- and intercellular vacuoles continued the dynamic development of the capillary network.

**Figure 3:**
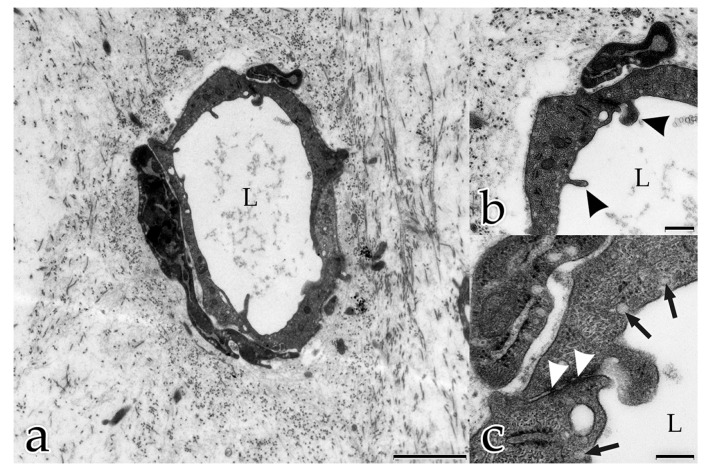
Coculture of ECs with FBs after 20 days: (**a**–**c**) cross section of a tube embedded in a densely organized ECM. Note the lumen (L); (**b**) magnification of (**a**) EC luminal surface with finger-like filopodia (arrowheads); (**c**) magnification of (**b**) tubular ECs overlap each other; they are connected by tight junctions (white arrowheads). Small vesicles and caveolae mirror pino- and exocytotic activities (arrows). (**a**) Bar: 2.500 nm; (**b**) Bar: 500 nm; (**c**) Bar: 250 nm.

Day 20: tubular ECs contained many small vesicles that, together with their membrane-bound caveolae, suggested transendothelial transport. The endothelial luminal surface was uneven and possessed finger-like filopodia with vesicles and caveolae that mirrored pino- and exocytotic activities (Figure 3a–c). Cellular viability and function was indicated by an abundance of organelles with intact morphology, for example, mitochondria with outer and inner membranes and rER.

#### 2.1.2. Capillaries with Basement Membrane

**Figure 4:**
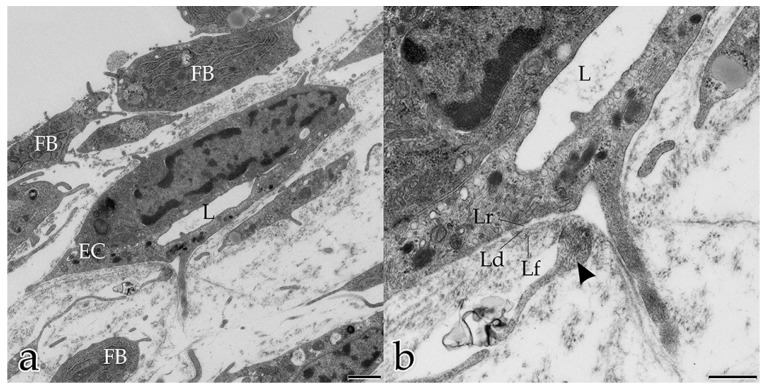
Coculture of ECs with FBs after 5 days: (**a**,**b**) cross section of a tube surrounded by FBs. Note the lumen (L). The BM surrounds the external surface of the endothelial tube; (**b**) magnification of (**a**): Note structures equivalent to the lamina rara (Lr), lamina densa (Ld), and lamina fibroreticularis (Lf) of the BM. Fibroblast protrusion (arrowhead) contacting the degrading BM as well as the sprouting EC. (**a**) Bar: 1.000 nm; (**b**) Bar: 500 nm.

After 5 days, the ECs had developed tubular structures, confirmed by the presence of tubular lumina in transverse and longitudinal ultrathin sections. Adjacent tube-forming ECs overlapped each other and were connected by tight junctions (Figure 3c). Similar to what seen in histological tissue sections, endothelial nuclei bulged towards the lumen in vitro. On their exterior surface, the endothelial tubes were surrounded by sheets of electron-dense material that were clearly distinguishable from the surrounding interstitial ECM and resembled the BM. The three parts of the BM, namely lamina densa, lamina rara, and lamina fibroreticularis, could be distinguished. The innermost sheet was a near-continuous layer closely associated with the tubule’s ECs. This lamina densa-like part of the BM aligned along the ECs and was separated from the cells by a narrow space corresponding to the developing lamina rara of the BM. The outermost sheet was a less dense but thicker layer of diffuse ECM around the tube, representing the lamina fibroreticularis (Figure 4a,b). In cross sections of tubules undergoing sprouting angiogenesis, degradation of the lamina rara and lamina densa (both layers form the basal lamina) was visible. Here, fibroblast protrusions extending into the angiogenic active sites and contacting the degrading BM as well as the sprouting ECs were observed (Figure 4a,b).

Days 10, 14, and 20: the endothelial tubular structures had developed both horizontally- and vertically-oriented tubes. Whereas the lumina of a few tubules were devoid of structures, other tubules contained amorphous material with cellular debris and vacuoles. BM material continued to differentiate into clearly separate layers.

#### 2.1.3. The Interstitial Extracellular Matrix: Its Role in Cellular Trafficking and Cellular Interaction

**Figure 5:**
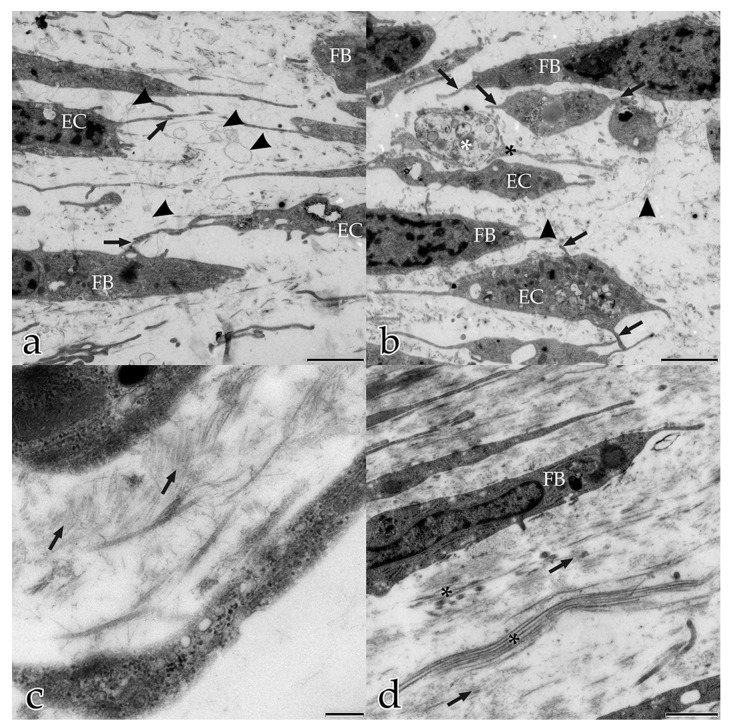
Coculture of ECs with FBs after 5 days (**a**,**b**), and after 20 days (**c**,**d**). In (**a**,**b**) FBs and ECs are interconnected with each other by their long filopodial processes (arrows). Fine ECM fibrils form a network between the cells (arrowheads). Extracellular vesicles (white asterisk) located within the ECM. Cellular filopodia contact the extracellular vesicles; (**c**,**d**) ECM with a close resemblance to a histological section of loose connective tissue in situ. Densely organized and well-differentiated meshwork of collagen bundles (asterisks), fibers, and fibrils (arrows). Thick collagenous bundles are associated with diffuse, poorly contrasted ECM elements. (**a**–**c**) Bar: 2.500 nm; (**d**) Bar: 1000 nm. Day 10: the in vitro construct was characterized by a more homogenous distribution of ECM that was most dense near the endothelial tubular structures. As observed after 5 days, fine fibrils had built up a connecting and now obvious scaffold network between the cellular components of the construct. This fine fiber net formed a framework for the organization and arrangement of the cells. It appeared that the angiogenic ECs used the ECM fiber net as a guiding structure for their migratory activities to align into tubular structures (Figure 2b).

After 5 days, the interstitial ECM was a randomly orientated, interlocking mesh of fibers and electron-dense molecules. FBs and ECs were dispersed within the ECM that they themselves had secreted. Both FBs and ECs were interconnected with each other by their long filopodial processes (FBs with FBs, FBs with ECs, ECs with ECs). Fine ECM fibrils built up a connecting network between the construct’s cells (Figure 5a,b). Round to ovoid extracellular vesicles surrounded by membranes were scattered within the interstitial matrix fibrils. Cellular filopodia of both cell types contacted and connected to these extracellular vesicles and even partially encircled them (Figure 5b). The cellular uptake and extrusion of membrane-coated vacuoles indicated trafficking activities within this dynamic web of interstitial ECM (Figure 1b,c).

Day 14: a more clearly defined network of interstitial ECM was present, and greater amounts of ECM progressively filled the intercellular spaces. Notably, adjacent to the endothelial tubes, the density of the interstitial fibers and the number of bonded electron-dense ECM molecules had increased greatly (Figure 4a). Many lamellae of fine fibrils surrounded the endothelial tubular structures. Longitudinal and transverse sections of thick parallel and obliquely orientated extracellular matrix structures, typical for mature collagen fibers, were associated with the fine fibrillar elements (Figure 5c).

**Figure 6:**
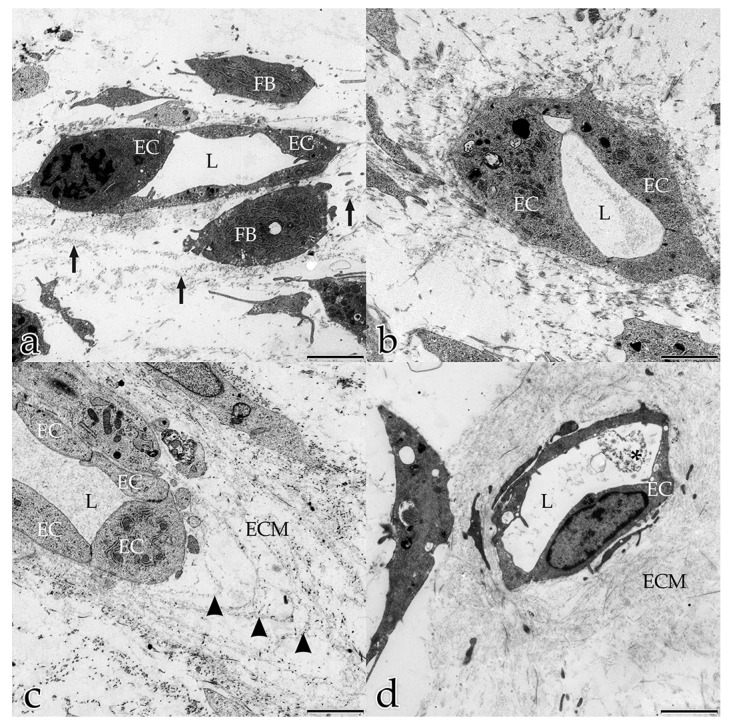
Coculture of ECs with FBs after 5 days (**a**); 10 days (**b**); 14 days (**c**); 20 days (**d**). EC tubular structures with lumina (L) in transverse sections: (**a**) the ECM presents as a randomly orientated mesh of fibrous and electron-dense molecules (arrows); (**b**) the ECM is most dense in the periphery of the endothelial tubular structures; (**c**) lamellae of fine fibrils surround the endothelial tubes (arrowheads) and build up a scaffolding network between the cells; (**d**) densely organized ECM, with a close resemblance to in vivo loose connective tissue, fill nearly all intercellular spaces. Vacuoles and parts of cellular membranes lie within the tubule’s lumen (asterisk). (**a**–**d**) Bar: 2.500 nm.

Day 20: the coculture constructs consisted of a densely organized ECM that had a close resemblance to histological tissue sections of loose connective tissue. The interstitial ECM filled nearly all intercellular spaces. The now regularly organized ECM had a dense, well-differentiated meshwork of collagen bundles, fibers, and fibrils that fully enmeshed all cells and tubular structures (Figure 6d). The collagen bundles were arranged orthogonally throughout the interstitial spaces. Capillary-like tubes were found embedded in the densest ECM within the construct. Thick collagenous bundles, associated with diffuse, poorly contrasted ECM elements (Figure 5c,d), surrounded the tubular structures.

#### 2.1.4. Monocultures of ECs and FBs

Over the complete period of observation (5 days until 20 days in vitro cultivation) the ECs in the monocultures adhered to the culture plate. The viability of the ECs was shown by their large number of well-preserved organelles; their nuclei and nuclear membranes were clearly visible and demarcated against the surrounding cytoplasm.

After 5 days, 10–15 layers of FBs were dispersed in an ECM secreted by the FBs themselves. Cellular densities in the upper and in the lower 3–4 layers were greater than in the intermediate layers. The interstitial ECM was densely aggregated in the outermost layers. After 10 days, the FB cultures consisted of up to 14–17 layers of cells within a loose meshwork of ECM. Overall, the cells and the interstitial ECM were evenly dispersed within the construct. In the more superficial strata, elongated FBs were more densely packed than elsewhere. Cells were separated by spaces with varying amounts of ECM material. The FB monocultures had built up to 15–23 cell strata by 14 days in vitro. We observed a more clearly defined arrangement of interstitial ECM and greater amounts of it progressively filling the intercellular spaces. Longitudinal and cross sections of thick parallel and oblique orientated collagen fibers arranged in both cell-dense and loose areas were seen. After 20 days, 20–23 strata of FBs were found to be embedded in an interstitial ECM that filled nearly all intercellular areas.

### 2.2. Morphology and Morphometry of Vascular Development and Extracellular Matrix Organization over the Timeline from 5 to 20 Days in Cocultures Assessed by Light Microscopy

After 5 days, ECs either aggregated into clusters or were arranged into tubular structures.

At this stage, the endothelial tubes were distributed evenly within the construct. Many fine cellular filopodia could be seen extending into the ECM. The endothelial tubes were characterized by single, double, triple, or multiple branches at their polar extremities. Yet, single branching tubes dominated.

At the same time, ellipsoidal to circular-shaped clusters of endothelial cell were found all over the cultures but were most common in the periphery and center of the culture plates. Originating from opposing poles, new endothelial sprouts arose from those clusters. Ongoing extension of the endothelial sprouts resulted in the elongation of the cellular clusters that ultimately transformed the clusters into capillary-like tubes. At this time, the majority of the capillary-like tubes had a characteristic central bulge with slender ends.

After 10 days, the number of tubes had decreased compared to day 5; this reduction continued until day 20 of coculturing (Table 1). In contrast, the length of tubes was greater at day 10 and continued to increase until day 20. Tubes had a uniform diameter along their entire length. The number of sprouting tubular branches was also higher at days 10 and 14, but by day 20 had dropped back to the same level as at day 5.

While the endothelial cell clusters disappeared from the peripheral areas, they remained in the middle of the cell culture dish.

After 14 days, the spreading of the endothelial tubes was maintained through the growth of single-, double-, and multibranched tubes. Sprouting angiogenesis originated all over the tubular structures with the sprouts extending across to neighboring tubes and forming a finely organized anastomosing tubular network while crossing the constructs’ general parallel organization. Networking occurred over the whole construct, but particularly in the periphery of the culture plate.

The cell clusters had largely disappeared after 14 days of coculturing.

After 20 days, further expansion and anastomoses had resulted in a denser tubular network with the highest number of branching endothelial tubes, mostly single- and multibranched endothelial tubes. Strikingly, low-branched endothelial tubes that were mostly found in the center of the construct, orientated themselves radially towards the peripherally-occurring multibranched tubes. Detailed measurements and standard deviations are summarized in Table 1.

#### 2.2.1. Quantification of Angiogenesis in Cocultures by Light Microscopy and Statistical Evaluation of the Morphometric Analysis

The endothelial tubes became significantly longer over the experimental timeline from 5 days (168.93 µm ± 8.19) to 20 days (475.46 µm ± 29.55, *p* = 0.021), however the tubular diameter did not change (*p* = 0.270). The number of tubes decreased from day 5 (9.35 ± 0.82 per mm^2^) to day 20 (2.19 ± 0.21 per mm^2^, *p* = 0.002), and, over the same period of time, the number of tubes with branches increased significantly with a maximum on day 14 (46.5 ± 10.01 per mm^2^), but fell to a minimum at day 20 (26.5 ± 8.19, *p* = 0.020). The length of the branches also increased significantly over time from day 5 (38.07 µm ± 6.23) to day 20 (190.16 µm ± 20.16, *p* < 0.001). Similarly, the percentage of multibranched endothelial tubes increased over time, from 10.0 ± 4.55% at day 5 to 39.5 ± 15.16% at day 20 *p* = 0.036. The pairwise comparisons (Bonferroni) of the culture time points revealed significant differences in the reduction of the number of tubes between day 5 and day 20 (*p* = 0.004), day 5 and day 14 (*p* = 0.020), as well as between day 10 and day 20 (*p* = 0.010). From day 10 until day 20, the length of the branches increased significantly. The decrease in the number of tubes was correlated with an increase in the length of the endothelial branches from day 5 to day 20 (*p* = 0.044, timeline: *p* = 0.002).

#### 2.2.2. Morphologic Analysis of EC and FB Mono Cell Cultures by Light Microscopy

After 5 days, the endothelial monocultures formed a monolayer of nucleated ECs of varying size that were adherent to the bottom of the culture dish. The ECs were polygonally shaped and had cytoplasmic projections interconnecting with neighboring cells. The cells had formed a subconfluent monolayer interrupted by a few large, empty spaces. After 10 days, the endothelial monocultures had developed a nearly confluent monolayer of polygonal- to spindle-shaped ECs. Within the monolayer, individual ECs had arranged themselves linearly side by side. After 14 days, the monolayer was closed. The formation of endothelial planar, circular structures (early stages of the angiogenic cascade) within the monolayer was observed. At day 20, single cell strands extended into a two-dimensional network of capillary-like structures, while the confluent monolayer still covered the culture dish.

Over a similar timeframe, monocultures of FBs built up a 3D multilayered cell construct that was adherent to the culture dish. Elongated, spindle-shaped, nucleated FB were densely aggregated and formed several vortices in the cell culture dish.

### 2.3. ECM Protein Localization and Quantification by Phase-Contrast Microscopy after 5, 10, 14, 20 Days of Culturing

**Figure 7:**
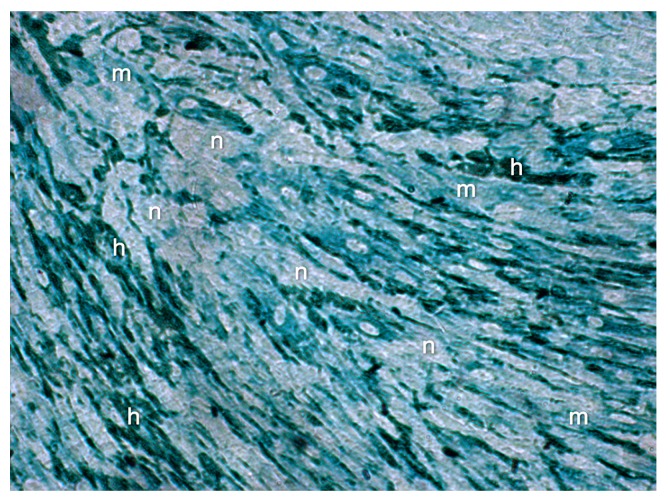
Immunolocalization of the ECM proteins collagen III, fibronectin, and laminin in cocultures of FBs and ECs after 14 days: the score for the immunolabeled color intensity ranges from high (**h**) to moderate (**m**) to negative (**n**). Magnification ×20.

**Figure 8:**
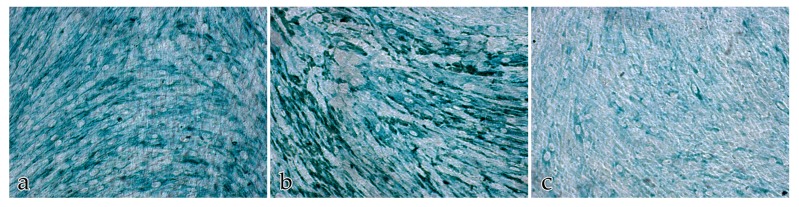
Immunolocalization of the ECM proteins collagen III, fibronectin, and laminin in cocultures of FBs and ECs after 14 days: (**a**) immunolocalization of collagen III; (**b**) immunolocalization of fibronectin; (**c**) immunolocalization of laminin. Magnification ×20.

**Figure 9:**
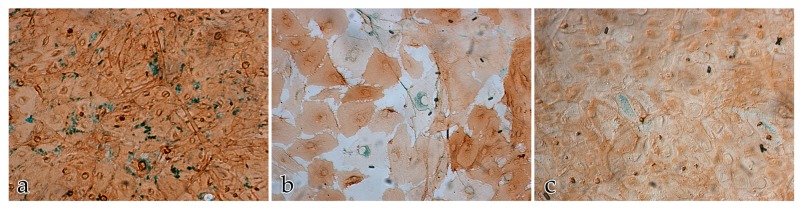
Immunolocalization of the ECM proteins (green) collagen III, fibronectin, and laminin in EC monocultures after 14 days. The ECs are immunolabeled with anti-CD31 (brown staining): (**a**) immunolocalization of collagen III; (**b**) immunolocalization of fibronectin; (**c**) immunolocalization of laminin. Magnification ×20.

**Figure 10:**
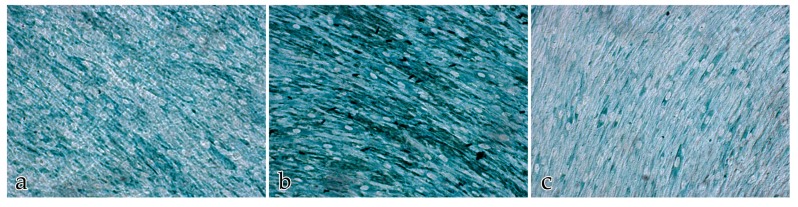
Immunolocalization of the ECM proteins collagen III, fibronectin, and laminin in FB monocultures after 14 days: (**a**) immunolocalization of collagen III; (**b**) immunolocalization of fibronectin; (**c**) immunolocalization of laminin. Magnification ×20.

Neither the buffer negative control nor the IgG negative control had a positive immunohistochemical response. The score for the immunolabeled color intensities and immunolocalization of the ECM proteins after 14 days is shown in Figure 7, Figure 8, Figure 9 and Figure 10.

#### Statistical Evaluation of the ECM Protein Measurements

*Total amount of immunolocalized ECM and intragroup differences over the timeline.* In general, both the cell culture system and the period of time had significant influences on the total amount of the immunolocalized ECM (cell culture: *p* < 0.0001, timeline: *p* = 0.006). Over the timeline from 5 days to 20 days, the total amount of visualized ECM differed significantly within the cocultures (*p* = 0.038) and within the EC monocultures. The total ECM within the cocultures was 36.21 ± 5.92% on day 5 and 23.40 ± 19.64% on day 10, and it underwent a statistically significant increase to 58.86 ± 0.97% by day 14, which was followed by a statistically significant decrease to 11.01 ± 2.73% (*p* = 0.010) by day 20. The cocultures showed significantly more total ECM than the EC monocell cultures (log 0.469) (*p* = 0.001) but did not differ with respect to FB monocell cultures (*p* > 0.999, log 1.477 and 1.405, respectively). Over the timeline from 5 days until 20 days, the total amount of ECM in FB monocultures was not significantly different from that of the cocultures (for details please check in Table 2). 

*Calculation of collagen III, fibronectin, laminin, and of the intergroup differences during the period of cultivation.* On day 5, no statistical difference in the proportion of laminin, fibronectin, or collagen III was found. At day 10, the difference in collagen III expression was nearly significant (*p* = 0.145), with FBs expressing 31.33 ± 1.62%, cocultures 40.60 ± 2.86%, and ECs 44.92 ± 7.92%. After 14 days of culturing, the amount of collagen III was significantly higher (*p* = 0.009) in ECs (65.85 ± 2.81%) than in the cocultures (46.57 ± 3.99%, *p* = 0.028) and FB cultures (36.74 ± 4.16%, *p* = 0.009). After 14 days of culturing, the expression of fibronectin was significant (*p* = 0.018). The FB cultures displayed a significantly higher amount of fibronectin-positive ECM (41.15 ± 0.53%) than the cocultures (37.59 ± 0.72%, *p* = 0.031) and the EC cultures (17.46 ± 6.87%, *p* = 0.020). Laminin expression was significant only after 20 days, (*p* = 0.016). In the EC cultures the amount of laminin was 39.32 ± 28.06%, which was significantly higher than in the cocultures (24.70 ± 12.5%, *p* = 0.016), but not higher than in the FB cultures (42.35 ± 9.07%).

The total amount of immunolocalized ECM significantly influenced the percentage of collagen III (*p* = 0.010) and of fibronectin (*p* = 0.014) by day 14. For collagen III, the amount of total ECM had a negative feedback from day 14: here, the proportion of collagen III decreased by a fraction of 0.412 with each increase in the amount of total ECM (confidence interval 0.165–0.659). For fibronectin, the amount of total ECM had a positive feedback from day 14: here, the amount of fibronectin grew significantly by 1 (from an average of 0.353) with the increase of ECM total amount (confidence interval 0.116–0.589, *p* = 0.014).

## 3. Discussion

The multifaceted composition and interplay of cells and molecules within a tissue or organ play an essential role and can only be mirrored in cocultures of at least two different cell species [13,23,24,25]. To optimize the existing in vitro models of angiogenesis [26,27], it is essential to take into account the interaction of the ECs with the cells and molecules present in their physiologic environment, and to include further cell types, especially stromal FB [13,24,25,28].

### 3.1. FBs Take an Active and Physical Part in the Angiogenesis Process of ECs

In our study, during the coculture of ECs with FBs, a microenvironment was generated that allowed and furthered cell–cell and cell–matrix interactions. In the early stages, both FBs and ECs possessed long filopodial processes that interconnected FBs with FBs, FBs with ECs, and ECs with ECs. FBs were seen frequently adjacent to the endothelial tubular structures, and physically contacted endothelial tubes at specific sites thereby initiating the degradation of the BM and the sprouting of a new vessel. These observations demonstrate that FBs take an active and physical part in the angiogenesis process of ECs. Fibroblasts are the main cells that secrete ECM components and growth factors, including VEGF-A, FGF, PDGF [6]. Our results suggest that FBs are not only passive bystanders that secret scaffold material and proangiogenic factors, but they also influence the activation of endothelial angiogenesis by their physical contact with ECs. Our observations of the dynamic involvement of FBs in angiogenesis suggest that angiogenesis models based on endothelial monocultures do not adequately explain in vivo intercellular communication [13,23]. The obvious influence of FBs must be considered when collecting and interpreting in vitro results.

### 3.2. Microvesicles in Intercellular Information Transfer

In the present study, several different mechanisms of intercellular communication were observed in the cocultured cells. To bridge short distances, cellular projections of adjacent ECs and FBs contacted each other. In addition, cellular material that was released by specific cells into the extracellular space appeared to be taken up by their neighboring cells.

This form of intercellular interaction was seen frequently in close proximity to tubular structures during the early stages of the morphogenesis of the in vitro constructs. As our morphological study presents no evidence for the exchange of material between the different cell species, further studies are planned to investigate this largely unknown phenomenon. In addition, in each developmental stage examined, extracellular vesicles surrounded by membranes were found to be scattered amongst the interstitial matrix fibrils, and cellular filopodia from both cell types were in contact with these extracellular vesicles. We hypothesize that these structures are microvesicles that were released by the cells, serving as vehicles for the transfer of angiogenic factors over longer distances [29]. Microparticles were found to be released in monocultures of ECs in an earlier study at the time of their integration from the monolayer into three-dimensional capillary-like structures [30]. It is only recently that the role of microvesicles in intercellular interactions has become a focus of research interest. Today, it is generally accepted that microvesicles mediate communication between cells in vivo [29,31,32]. After their synthesis, microvesicles are released via exocytosis and float within the extracellular space to interact with an adjacent cell in a paracrine manner. Vesicles deliver information to their recipients by binding to specific receptors [33]. Recent studies report that extracellular vesicles play a role as proangiogenic mediators [33,34,35]. It has been suggested that angiogenic extracellular vesicles are released from different types of cells, and that their content ranges from lipids, to proteins, to mRNA [33,34,35]. Interestingly, a cargo of microvesicles is also involved in the early steps of the angiogenic cascade. Soucy and Romer [36] described endothelial vesicles that carry MT1-MMP, i.e., the MMP responsible for the degradation of the BM in the beginning of sprouting angiogenesis. Using transmission electron microscopy, we observed that the ECs took up extracellular vesicles and that the capillary lumen formed by fusion of those vesicles. Our results mirror the dynamics of lumen formation in vivo and are supported by the observations from Kamei et al. [18], who reported lumen formation by intracellular and intercellular fusion of endothelial vacuoles in live animals. They used time-lapse imaging to examine the dynamics of endothelial vacuoles and their contribution to vascular lumen formation [18].

### 3.3. The Vascular Structures and the ECM of Cocultures Mirror the Topography of Histological Tissue Sections

In vivo, the fibrillary and nonfibrillary interstitial ECM milieu, in which blood capillaries are embedded, is rich in fibrillar and nonfibrillar collagens, elastic fibers, glycosaminoglycans, and glycoproteins, such as fibronectin, laminin, and other molecules [3]. Our ultrastructural observations revealed that the interstitial ECM progressively filled the spaces between the cells over the investigation timeline (day 5–day 20), until the in vitro construct consisted of a densely organized ECM that formed a framework for the organization of the cells and displayed a close resemblance to histological sections of loose connective tissue.

We examined the expression pattern of the three different ECM components collagen III, fibronectin, and laminin, which are known to be relevant in different steps of angiogenesis [6,7]. In our study, the amount of immunolocalized ECM differed significantly over the observation timeline as it followed an undulating pattern with the total ECM being 36.21 ± 5.92% at day 5, followed by a decrease to 23.40 ± 19.64% by day 10, then an increase to 58.86 ± 0.97% by day 14, and a final decrease to 11.01 ± 2.73% (*p* = 0.010) by day 20. This pattern mirrors ECM development and maturation over time as analyzed by the pixel intensity profile. Ultrastructurally, its configuration and accumulation started from a randomly organized structure finally maturing into a homogenously distributed matrix consisting of smaller but more condensed molecules over the cultivation timeline. Our results corroborate the possibility that, similar to the in vivo situation, the ECM produced in the coculture assays is actively remodeled by FBs [25] and changes its composition in order to adjust to the individual developmental demands of the tissue. Notably, matrix remodeling FBs are crucial in vascular development through transmitting biochemical signals and mechanical forces affecting cell survival, cell shape, and cell orientation [19]. Therefore, it is possible that the ECM that we immunolocalized was replaced by other ECM components during the maturation process. In addition, our studies, comparing the cocultures with the monocultures of FBs and ECs, support that the ECM is mainly synthesized by the FBs.

In our cocultures of ECs with FBs, the three-dimensional ECM was synthesized by the cells themselves. This ECM not only served as a scaffold for the vascular structures, but it also played an active role in the communication between cells involved in angiogenesis. This confirms observations by Neve et al. [3], who reported that the ECM consists of and stores a multitude of proteins, glycoproteins, and polysaccharides that act as proangiogenic or antiangiogenic factors controlling vascular development and remodeling [3].

### 3.4. Molecules and Fibers of the ECM Guide the ECs during Angiogenesis

At the onset of coculturing, FBs and ECs were distributed randomly. The ECM had built up a connecting network of very fine fibrils between these cells. We assume that these fine fibers, running from cell to cell and connecting them with each other, may be fibronectin. Soucy and Romer [36] described that a characteristic of fibronectin is its configuration into fine fibrillar structures, and that fibronectin initiates the matrix organization into its typical architecture [36]. This is supported by our observations that the fine fiber net not only dictated the position of the cells within the cocultures, but also constituted a framework for other ECM components, including collagen fibrils. We also observed that the ECs lined up with other ECs using the fine ECM fiber net to guide their migratory activities. In this context, it is important to know that ECs selectively adhere to fibronectin [36].

In our study, the expression of fibronectin was found to decrease significantly after 10 days of cocultivation. In vivo, fibronectin is strongly associated with the periphery of developing vessels, but it is only weakly expressed in the mature vasculature [6,37]. The decrease of fibronectin found in this study can be interpreted as an indication of the maturation of the capillary-like structures. Hetheridge et al. [25], who also cocultured ECs with FBs, reported that the growth of tubes in vitro ceased after 14 days. At the endothelial intercellular junctions, they measured an increase in vascular endothelial cadherin, which is known to suppress the sprouting of new tubes [25].

Collagen III is a fibrillary protein that plays an important role in stabilizing blood vessels and affects fibroblast function [7]. Our observations showed an increasing amount of immunolabeled collagen III from day 10 to day 20 in parallel to the appearance of differentiated fibrillar structures within the ECM.

### 3.5. Formation of A Three-Layered BM In Vitro

Sheet-like organized ECM components surrounded the tubular structures on their external surface and resembled the BM. The formation of a BM synthesized by both ECs and FBs proceeded with the ongoing maturation and stabilization of a new vessel [9]. The BM typically consisted of an electron-dense layer (lamina densa) separated from the plasma membrane by an electron-lucent layer (lamina rara). In monocultures of ECs, a BM consisting of several layers mimicking the in vivo situation did not develop [38]. The BM is multifunctional and important for endothelial luminal polarization. The ECs in the capillary-like structures that developed in our cocultures clearly showed a correct polarity with nuclei bulging towards the lumen. EC nuclei orientated towards the “wrong” abluminal side are an artefact that occurs frequently in monocultures of angiogenic ECs [38].

Laminin is an important molecule of the BM [3,7] and, together with fibronectin, forms networks within the BM contributing to BM stability [3,7]. In our study, laminin was evenly expressed over the period of observation. In monocultures of ECs the amount of laminin was significantly higher than in the cocultures. Whether this is an effect of the absence of FBs can only be speculated.

### 3.6. Signs of Maturation of Capillary-Like Tubular Structures In Vitro by Day 20

In summary, the following signs indicate maturation of capillary-like tubular structures in the in vitro constructs by day 20. Firstly, the interstitial ECM presented a dense, well-differentiated meshwork of collagen bundles, fibers, and fibrils that fully enmeshed all cells and tubular structures. Secondly, tubular structures were build up by ECs that were connected to each other by tight and adherens junctions. Thirdly, the endothelial tubes were surrounded by a BM that had differentiated into three clearly separated layers. Fourthly, individual fibroblastic cells with small amounts of rER were detected, indicating their differentiation into fibrocytes. Lastly, the morphometric analysis revealed a decrease in the number of tubes and an increase of multibranched endothelial tubes, as well as an increase in the length of endothelial branches, indicating that remodeling and maturation of the vascular network in vitro had occurred.

In conclusion, our study underlines the importance of morphological and ultrastructural investigations that should proceed hand in hand with molecular analyses.

## 4. Materials and Methods

### 4.1. Cells and Culture Conditions

Human dermal microvascular ECs from neonatal foreskin (HMVEC-D; Lonza, Walkersville Inc., Walkersville, MD, USA) were cultured according to the manufacturer’s instructions in basic EC growth medium 2 (EGM-2-MV, Lonza, Switzerland) supplemented with fetal bovine serum (FBS), fibroblast growth factor (rhFGF), vascular endothelial growth factor (VEGF), vitamin C, GA-1000 (gentamicin/amphotericin B), and hydrocortisone. Human juvenile foreskin fibroblasts (FB) were isolated from residual tissue of circumcision surgery (with patient’s ethical consent and permission from Charité, Berlin, Germany EA1/081/13) and cultured in Dulbecco’s Modified Eagle’s Medium (DMEM) containing 10% FBS, 1% l-glutamin, and 1% penicillin/streptomycin (10,000 U/mL; all from Sigma-Aldrich, Taufkirchen, Germany). All cells were maintained at 37 °C in a humidified atmosphere (37 °C, 5% CO_2_). The cell culture medium was changed every 2–3 days. For each experiment, HMVEC-D were used at passage 5, and FBs were used at passages 9–10. The bottom of the culture wells was not coated with gelatin or other substances.

### 4.2. Priming of Fibroblasts

Adaptation (priming) of the FB monocell cultures to the EC medium was achieved in 48 h steps. The fresh FB medium was first mixed with 25% of EC medium, after 48 h with 50%, after further 48 h with 75%, and finally with 100% of EC medium.

### 4.3. Direct Cocultures of ECs with FBs and Monocultures of ECs or FBs

For cocultures, adapted FBs (5.000 cells per well) were seeded on a 24-well plate and cultured for 10 days. After this period of time, 20.000 ECs were seeded on top of the FBs for direct cellular contact and incubated for another 5, 10, 14, and 20 days in EC medium, which was replaced every 2–3 days (modified assay according to Richards and Mellor, 2016). After 5, 10, 14, and 20 days of culturing, the cells were fixed and processed as described in Section 4.4 and Section 4.5.

The monocultures of ECs or FBs were cultivated separately to assess and compare their cell behavior, morphometric features of angiogenesis, and extracellular matrix production. The monocultures were fixed and processed after 5, 10, 14, and 20 days of culturing, as described Section 4.4 and Section 4.5.

### 4.4. Transmission Electron Microscopy of the Cocultures

Cocultures for transmission electron microscopy (TEM) were grown on transwell membranes with a pore size of 0.4 µm (Corning, Tewksbury, MA, USA) for 5, 10, 14, and 20 days. The cultures were washed with 0.1 M cacodylate buffer (cacodylic acid sodium salt trihydrate, Roth, Karlsruhe, Germany) and fixed for 1 h at 4 °C in Karnovsky’s fixative (Merck Eurolab, Darmstadt, Germany). After fixation, the cells were postfixed in 1% osmium tetroxide (Chempur, Karlsruhe, Germany) in 0.1 M sodium cacodylate buffer for 1 h. Dehydration followed through a graded series of ethanol. The specimens were embedded in a mixture of Agar 100 (epoxy resin), DDSA (softener), MNA (hardener), and DMP 30 (catalyst) (all: Agar Scientific, Stansted, Great Britain, UK). The polymerization was done at 45 °C and 55 °C, each for 24 h. Semithin sections (0.5 µm) were cut with an ultramicrotome Reichert UltracutS (Leica Microsystems, Wetzlar, Germany) and stained with modified Richardson solution [39] for 45 s on an electric hotplate at 80 °C. The examination of semithin sections was carried out using a light microscope (Olympus CX21, Olympus, Stuttgart, Germany). Next, ultrathin sections were cut with the UltracutS. Sections of 70 nm were mounted on nickel grids (Agar Scientific, Stansted, Great Britain, UK), stained with 2% uranyl acetate, stabilized by lead citrate Ultrostain 2 (Leica, Wetzlar, Germany), and examined to detect the occurrence of capillary structures, cell–cell contacts, cell organelles, and cell shapes as well as to analyze ECM organization, distribution, and differentiation using an EM109 electron microscope (Zeiss, Jena, Germany). Photographs were taken and processed using an Adobe Photoshop Program (Adobe System, Unterschleissheim, Germany).

### 4.5. Immunocytochemistry

Cells were washed with PBS, fixed with methanol/acetone (1:1) at −20 °C, and air-dried for at least 1 h. Afterwards, the cells were fixed for a second time with 4% formalin diluted in PBS overnight at 4 °C and then washed briefly with tap water and TBS. After antigen demasking with Target Retrieval Solution pH 9.0 (DAKO, Glostrup, Denmark) for 30 min at 86 °C and immersion in Protein Block Serum-Free (DAKO Diagnostika, Hamburg, Germany), the cultures were immunolabelled. For specific EC identification, mouse anti-CD31 (1:50, clone JC70A, DAKO, Glostrup, Denmark) was employed. To assess ECM protein distribution, mouse anti-human fibronectin (1:50, clone A-11, Santa Cruz, Heidelberg, Germany), rabbit anti-human collagen III (1:50, Abcam, GBR, Cambridge, UK), and rabbit anti-human laminin (1:50, DAKO, Glostrup, Denmark) were used. After 2 h incubation with CD31 at room temperature, cells were washed with TBS and incubated with anti-mouse Polymer EnVision System: HRP (DAKO, Glostrup, Denmark) for 30 min at 37 °C. After PBS wash, HRP was visualized by diaminobenzidine (Sigma-Aldrich, Taufkirchen, Germany) for 20 min at 37 °C. To assess ECM proteins, an overnight incubation with the different antibodies was performed at 4 °C. The antibodies had been diluted earlier in 3% bovine serum albumin (BSA) (Roth, Karlsruhe, Germany), 2% goat normal serum (GNS) (DAKO, Glostrup, Denmark), and incubation buffer (with 1% milk solely for rabbit antibodies) for 30 min at room temperature. Antibody incubation was followed by a TBS wash and the addition of anti-mouse Polymer EnVision System-HRP (DAKO, Glostrup, Denmark) and donkey-anti-rabbit-Ig-HRP (Linaris, Dossenheim, Germany), respectively, for 30 min at 37 °C. Negative controls were performed, in which the primary antibody was replaced by buffered anti-rabbit IgG (1:500, DAKO, Glostrup, Denmark) or anti-mouse IgG1 and IgG2b (1:25, both DAKO, Glostrup, Denmark). Subsequently, the cells were washed with PBS and treated with HistoGreen (Linaris, Dossenheim, Germany) for 10 min at room temperature for HRP detection.

### 4.6. Morphometric Analysis and Quantification of Angiogenesis

Four wells from each day (5, 10, 14, and 20) and culture type (cocultures of ECs with FBs, monocultures of ECs or FBs, respectively) were examined with a light microscope (Zeiss MicroImaging GmbH, Jena, Germany) and photographed with a digital camera (ColorView, Olympus, Münster, Germany). PC-based laboratory imaging programs (analysis docu, Version 5.2, Olympus, Münster, Germany and NIS-Elements AR, Version 4.5, Nikon, Düsseldorf, Germany) were used for processing. For measurements of capillary-like structures, visual fields were standardized by taking 7 images of each cell culture at the same magnification (5×) with a total area of inspection of 40.6 mm^2^. The morphometric features of capillary-like structures that were evaluated included: average number of tubes per mm^2^, average length of tubes (10 tubes per field of view), average diameter of tubes (10 tubes per field of view), average number of tubes with branches, including the percentage proportion of branching and the average length of branches (10 branches per field of view), and branching distribution pattern (single-, double-, triple- and multibranched). Branches were defined as projections of capillary-like structures, which were detectable at a magnification of 5×. The percentage proportion of branching was defined by the number of branched tubes in relation to the total number of tubes that were counted.

### 4.7. Quantitative Area Percentage Measurement for the Assessment of ECM Immunolocalization

The assessment of immunolabeled ECM components including the three proteins laminin, collagen III, and fibronectin, was conducted in cocultures and in monocultures after 5, 10, 14, and 20 days in vitro. Ten images were taken with a 20× objective from each cell culture type (Zeiss MicroImaging GmbH, Jena, Germany and ColorView, Olympus, Münster, Germany). The quantification of the fixed, immunostained ECM proteins was evaluated using a quantitative multiphase analysis for determining phase composition (analySIS docu, Version 5.2, Olympus, Münster, Germany) with a corresponding pixel intensity profile that was calibrated manually. We defined the color intensity ranges (negative, moderate, and high) for the separate phases. The proportion of positively labeled ECM (regions that displayed a distinct color change) was expressed as a percentage of the total area. The total amount of ECM was defined as the percentage of all positively labeled areas of one slide (moderate and high intensity). The amount of the individual ECM proteins (laminin, collagen III, and fibronectin) was determined as the proportion of the total response. The ECM production was measured through the pixel intensity profile, and the percentage of positively labeled pixels mirrored the quantity of immunolabeled ECM.

### 4.8. Statistical Analysis

Statistical analysis was performed by using IBM SPSS 24 (IBM Deutschland GmbH, Ehningen, Germany). Linear mixed regression models were conducted to determine the effect of the different wells and plates on the morphometric features of angiogenesis. In these models, (incubation) time was the fixed effect and the plate the random effect. Separate models were adapted for the following dependent variables: the average number, the length or diameter of the tubes respectively, the length of the branches, and the percentage proportion of branching over a set period of time. The Intraclass Correlation Coefficient (ICC) was defined as the percentage of variance due to differences between plates. A high ICC meant that there was a large variance between the plates, and thus the plate had a large influence on the result. In contrast to a fixed factor, the ICC generally refers to all plates and not distinctly to the plates that were used. Although the ICC values were high (between 22% and 65%), they were not statistically significant. Considering this, we utilized linear mixed regression models with Bonferroni correction for further uni- and multifactorial morphometric analyses. All the morphometric features of angiogenesis listed in 2.6 were tested for significant differences between the time points after 5, 10, 14, and 20 days of culturing, with particular focus placed on the time dependency and the association of morphometric variables. Bonferroni pairwise comparisons were conducted between each time of investigation.

All ECM parameters quantitatively analyzed were investigated by One Way ANOVA. All dependent variables (total amount of labeled ECM proteins, laminin, collagen III, or fibronectin, respectively) were tested with the independent variables time and cell culture type (cocultures of ECs with ECs, monoculture of ECs or FBs), the remaining ECM parameters, as well as the interactions between time and cell culture types. As the values of total ECM amounts were not normally distributed, we used logarithmic values for the regression models. Tukey’s (all pairwise) and Dunnett’s (all against the reference group day 20) post hoc test as well as Bonferroni correction adjustment in multiple comparisons were used in this model. To find out if there was a positive or negative feedback between the total ECM amount and specific ECM components, the influence of determining factors, collagen III, fibronectin, and laminin was tested against the total amount of ECM that had been immunolocalized. 

The results were considered statistically significant at *p* < 0.05. All data were reported as the mean values and standard deviation.

## Figures and Tables

**Table 1:** Angiogenesis measurements. Data as mean ± standard deviations; s = single; d = double; t = triple; m = multi-branched.

Cultivation Time (Days)	5	10	14	20
Number of tubes (per mm^2^)	9.35 ± 0.82	6.43 ± 1.27	4.91 ± 0.24	2.19 ± 0.21
Length of tubes (µm)	168.93 ± 8.19	339.39 ± 72.55	445.89 ± 63.47	475.46 ± 29.55
Diameter of tubes (µm)	12.01 ± 1.52	13.36 ± 0.93	12.14 ± 1.14	10.72 ± 1.14
Tubes with branches	28.00 ± 2.16	43.50 ± 14.18	46.50 ± 10.01	26.50 ± 8.19
Length of branches (µm)	38.07 ± 6.23	63.56 ± 12.93	107.41 ± 18.20	190.16 ± 20.16
Percentage portion of branches (%)	7.50 ± 1.00	17.50 ± 6.81	23.25 ± 4.27	29.75 ± 8.50
Branching distribution pattern (%)	s: 61.25 ± 14.68	s: 56.00 ± 2.71	s: 35.50 ± 6.95	s: 44.25 ± 9.88
	d: 22.75 ± 9.71	d: 23.50 ± 3.87	d: 24.50 ± 6.61	d: 22.25 ± 5.97
	t: 6.00 ± 7.66	t: 7.75 ± 5.56	t: 6.50 ± 2.89	t: 5.00 ± 4.40
	m: 10.00 ± 4.55	m: 12.75 ± 5.19	m: 33.50 ± 9.61	m: 39.50 ± 15.16

**Table 2:** Percentages of total ECM as well as of collagen III, fibronectin, and laminin production in cocultures of fibroblasts and endothelial cells and in mono cell cultures of fibroblasts and endothelial cells at 5, 10, 14, and 20 days. Data as mean ± standard deviations; CC = cocultures; EC = endothelial mono cell cultures; FB = fibroblast mono cell cultures.

Cultivation Time (Days)		ECM Total Amount%	Collagen III Proportion%	Fibronectin Proportion%	Laminin Proportion%
5	CC	36.21 ± 5.92%	47.90 ± 33.13%	32.84 ± 6.4%	19.26 ± 26.73%
	EC	5.90 ± 2.3%	29.86 ± 14.35%	36.94 ± 10.14%	33.19 ± 4.21%
	FB	41.73 ± 18.56%	38.09 ± 13.41%	29.42 ± 2.17%	32.49 ± 11.24%
10	CC	23.40 ±19.64%	40.60 ± 2.86%	37.31 ± 14.3%	22.09 ± 17.16%
	EC	1.85 ± 1.24%	44.92 ± 7.92%	23.02 ± 3.88%	32.07 ± 4.04%
	FB	23.66 ±16.75%	31.33 ± 1.62%	40.07 ± 2.64%	28.60 ± 1.02%
14	CC	58.86 ± 0.97%	46.57 ± 3.99%	37.59 ± 0.72%	15.84 ± 4.71%
	EC	3.14 ± 0.44%	65.85 ± 2.81%	17.46 ± 6.87%	16.68 ± 4.06%
	FB	58.41 ± 21.47%	36.74 ± 4.16%	41.15 ± 0.53%	22.12 ± 4.69%
20	CC	11.01 ± 2.73%	66.35 ± 12.08%	24.70 ± 12.5%	8.95 ± 0.42%
	EC	3.82 ± 3.94%	28.44 ± 22.23%	39.32 ± 28.06%	32.24 ± 5.83%
	FB	18.48 ± 10.41%	36.51 ± 11.59%	42.35 ± 9.07%	21.14 ± 2.52%

## Abbreviations

ECM	Extracellular matrix
3D	Three-dimensional
BM	Basement membrane
EC	Endothelial cell
VEGF	Vascular endothelial growth factor
MT1-MMP	Membrane-type 1 matrix metalloproteinases
FB	Fibroblast
CC	Coculture
mRNA	Messenger ribonucleic acid
RNA	Ribonucleic acid
HMVEC-D	Human Dermal Microvascular Endothelial Cells
EGM	Endothelial Cell Growth Medium
MV	Microvascular
rhFGF	Recombinant human basic fibroblast growth factor
GA	Gentamicin/amphotericin
DMEM	Dulbecco’s Modified Eagle’s Medium
TEM	Transmission electron microscopy
DDSA	Dodecenylsuccinic anhydride
MNA	Methylnadic anhydride
M	Mol
DMP	2,4,6-Tris(dimethylaminomethyl)phenol
EM	Electron microscope
PBS	Phosphate buffered saline
TBS	Tris-buffered saline
CD31	Cluster of Differentiation 31
HRP	Horseradish peroxidase
BSA	Bovine serum albumin
SPSS	Statistical Package for the Social Sciences
ICC	Statistical Package for the Social Sciences
BB3R	Berlin-Brandenburg Replace Reduce Refine
BMBF	Bundesministerium für Bildung und Forschung
